# Sketch of the Present State of Medicine, and of Medical Institutions, in Russia

**Published:** 1837-07

**Authors:** George Lefevre

**Affiliations:** late Physician to the British Embassy at St. Petersburg, &c.


### PART FIFTH.

### Medical Intelligence.

---

### SKETCH OF THE PRESENT STATE OF MEDICINE, AND OF MEDICAL INSTITUTIONS, IN RUSSIA.

By George Lefevre, m.d., late Physician to the British Embassy at St. Petersburg, &c.

### PART II.

### OF THE MEDICAL PROFESSION AND MEDICAL INSTITUTIONS.

### I. CLASSIFICATION OF THE MEDICAL PROFESSION.

It is now generally allowed that there is no essential distinction between medicine and surgery, although practitioners of medicine may be divided into different ranks and classes. Perhaps there is no country where this indivisibility, as regards the practice of the profession, is more strictly maintained than in Russia. The division of medical labour, which in former times simplified the duties of the respective practitioners in England, has never been adopted in Russia, where a physician embraces all the branches of the healing art, and acts as physician, surgeon, and accoucheur. If he do not practise his profession so generally as here stated, there is nothing to prevent him from using his own discretion in such matters. The physician in Russia is, in fact, identical with the general practitioner in England, save and except that in no instance does he dispense his own medicines. I employ the term general practitioner, because in reality the apothecary, at least in the English acceptation of the word, does not exist in Russia. Persons bearing this designation are mere venders of drugs and licensed preparers of physician's prescriptions: they are not supposed to be acquainted with more than the nocuous and innocuous properties of the drugs in their rough state. In no case are they allowed to compound medicines, or even sell the most innocent drug, without a written order from a medical practitioner. Their shop, or *Apteka*, is no laboratory; for they are not wholesale chemists and druggists, but mere makers-up of prescriptions.

Many of the English physicians in Russia formerly persisted in adhering to the customs of their mother-country, as to the division of labour in the profession. In some instances, however, even the most orthodox have waived this point of etiquette. Dr. Dimsdale tried the experiment of inoculating the Empress Catherine for the small-pox; which same operation Dr. Halliday performed on all the Emperor Paul's children, though unquestionably this task appertained to the province of the surgeon.

This combination of medical duties is not now a matter of choice, it is one of necessity, inasmuch as the interests of the mass of the community are to be considered of more importance than the ideas which a few individuals may form to themselves of medical orthodoxy; and in the present day all the English physicians residing in Russia are, as already stated, *de facto* general practitioners.

Although in a practical sense, then, there is little difference in the duties performed by the different practitioners, of whatever rank they may be, still the superiority of rank is, in point of etiquette, most rigidly enforced in all cases where form is allowed to exercise its prerogative. There are several classes of practitioners, but the deference paid to each is not in a ratio with their medical, so much as their military or civil ranks. The degrees conferred by the universities are the following:—Physician; Surgeon in chief; Surgeon in ordinary; Staff surgeon; Surgeon's mate; Hospital mate; Barber surgeon; Apothecary.

In general practice, there is no positive distinction in the labours allotted to the three first ranks. The physicians and the surgeons, in most cases, practise indiscriminately all branches of the profession. If they hold official situations in the military or civil service, then the difference of rank becomes immediately sensible. The physician receives homage from the surgeons, takes precedence as he passes through the wards of the hospital, signs documents, and makes valid his rank by several operations. The hospital mates, dressed in military uniform, march up and down the wards, halt, face about, and stand attention, as their superiors command them; for all society in Russia is divided into ranks and classes, and medical men are included in this category. Schlatzer informs us that, in 1781, "a physician ranked with a major in the army, and, as such, could drive four horses to his carriage; whilst those of inferior rank could drive only two An apothecary at court had the rank of captain, and his apprentices the rank of ensigns. The surgeons of the district had the rank of lieutenants." In the present day, physicians are ranked in the eighth class of the nobility, and surgeons in the seventh.

There are few instances, perhaps, where any practical use or abuse of such a division can be allowed to operate, but in some cases the possibility may exist. It is almost a law of the realm that no individual holding superior rank, whether civil or military, can be in the wrong in case of dispute with an inferior; and the spirit of this law is not without its influence in medical concerns. The superiority here alluded to is the honorary rank conferred by the crown, and not the medical one granted by the universities. Two physicians, of equal standing in medical honours, may be widely separated in civil or military rank; and he who bears no insignia of the order of St. Anne or St. Vladimir must give way in consultation to him whose breast is adorned with stars or crosses.

The physician, when called to the service of the imperial person, the *Leibmedicus*, or body physician, takes precedence of all his brethren, and his opinion must prevail in all cases where there is any official duty concerned. In the former part of this sketch it was mentioned that one of the Czars, suspecting that the illness of an illustrious individual was rather to be construed into a disinclination to appear at court, sent his body physician to ascertain the fact. Very similar proceedings take place in the present day. Patients, attended by ordinary physicians, are occasionally visited by body physicians, in order that they may enjoy every possible medical advantage, and this frequently without their request.

The hospital mates and surgeon's mates are completely under military control, although attached to civil institutions. They are subjected to punishments of various kinds for any impropriety of conduct.

The apothecaries, as before observed, are not to be considered as the same class of men who are so denominated in England. They are mere venders of drugs and preparers of recipes, and their shops are all licensed by government.

The first court *Apteka* was founded by an Englishman of the name of Frenshman, who arrived in Russia in 1581. He is reported to have brought with him an immense quantity of drugs; but it was some time before the natives understood the necessity of having recourse to medicine. The high price at which drugs continue to be sold in Russia would almost be proof sufficient that the aptekas were established by one of our countrymen; for, if apothecary's gain be synonymous with exorbitant charge in England, it is equally so in Russia. There is no country in which medicines are so highly charged; indeed, the price is almost double what it is in England. The apothecary's charge in Great Britain is not supposed to be in relation to the cost of the material; the value of his prescription is embodied in his draught, and is the means of his remuneration for his time and talents. The matter is very different in Russia, where the apothecary is the compounder only, and should be satisfied with a fair profit upon the material, as the prescription has already been paid for; whereas, the prices he pleases to put upon it allows of no such consideration on his part. It is true that there is a tariff for the price of medicines, to which he is compelled to adhere; but this is excessively high, and not regulated by the current prices of the drugs imported into the empire. The proprietors of these aptekas are almost all Germans.

The regulations, which were promulgated by the court physician, Dr. Blumentrost, in the time of Peter the Great, have, with very slight alterations, been handed down to the present time, and many of these are very excellent in their kind. To prevent mistakes arising from an imperfect knowledge of the Latin language, it was ordered that all prescriptions should be translated into Russian, and deposited in the chancery. The name of the physician and of the patient, together with the date of the day and month, were also registered in a book. The necessity of translating the prescription is now abandoned, but all the recipes are deposited in a drawer, and are not allowed to be given back; notwithstanding that each is copied into a daybook, which is subjected to the inspection of proper authorities, who pay regular visits to the aptekas for the purpose of examining the nature of the recipes. Not only are the original prescriptions not allowed to be returned, but a repetition of the same medicine cannot be procured without the physician's signature. A copy only is permitted to be issued from the apteka. From the same conservative principles, wholesale dealers in drugs are not allowed to sell them in small quantities. No medicine is allowed to be dispensed without being sealed with the private seal of the owner of the apteka, or with the seal of his establishment; for each shop is licensed under some specific name, generally taken from the name of the street in which it is situated; and the number of these is limited. This limitation in the number dates also from a remote period, and proceeded from good intentions; for it was asserted that the number of aptekas should be in a ratio with the demand for the medicines, and that, on the one hand, there should be a sufficient competition to prevent the public from being overcharged, and, on the other, not so great a supply as to allow the drugs to remain too long on hand, and be spoiled by age.

It is difficult to legislate in such matters; but the practitioner in Petersburg will find the advantage of sending his prescriptions to the aptekas which are in vogue, for the same recipe prepared in a frequented shop, or in one not much frequented, may show two very different things.

If poisons are prescribed in any dangerous quantity, the physician is compelled to sign his name in the *poison-book,* and state for what purpose he has prescribed the poison.

When these establishments were first instituted, they were all under the direction of an officer of state, who was styled the *Apteka Bojar.* They are now under the superintendence of a Chancery (*Physicant*), which is a court of appeal and punishment. If a physician can prove that any mistake has been committed in the preparation of his recipe, or that the drugs are not of good quality, it is the apothecary who suffers. If he can prove that the physician has made a mistake in the writing of the prescription, the apothecary is at least absolved, unless he magnanimously consent to take the blame upon himself. If the shop-boys are in fault, they are subjected to corporal castigation.

The last class is the *Tsirulnik*, or barber surgeon, and is a numerous and thriving brotherhood. It falls to their lot to bleed, cup, draw teeth, apply leeches, and perform other little jobs of minor consequence. They are well paid for their trouble: five roubles, or 3*s.* 6*d.*, is the common fee for their operations, and they are in great request.

There are no distinct aurists; aural surgery forming part of the practice of the ordinary surgeons. There are likewise but few oculists who devote their whole time to the diseases of the eye. Many physicians and surgeons include the treatment of these diseases in their general practice.

Dentists abound in every street, and their profession is perfectly distinct, and not within the pale of the medical faculty. There are no distinct chiropodists in Russia.

### II. REMUNERATION OF THE MEDICAL PROFESSION.

This subject allows of several divisions; viz. the pay of medical officers serving under government, either in the army or navy, in civil institutions, and in the universities; and the remuneration of private practitioners.

In regard to the remuneration of the private practitioners, it may be summed up in a few words,—it is almost optional with the patient. He pays in a ratio with his means, with his liberality, or with the estimation in which his attending physician is held by the court or by the public. In this respect custom has not fixed any regular fee; but the law obliges a medical man to attend any patient who demands his services, for which the practitioner can claim a fee of five rubles (3*s.* 6*d.*) per visit. This is an extreme law, which however has, within my knowledge, lately been enforced. In most instances, private attendance is paid for at the termination of the case. The payment is often in the shape of presents, as snuff-boxes, diamond rings, and plate of various kinds. It is by many considered not quite delicate to offer a physician money, though few would consider themselves aggrieved by this species of remuneration.

The more common mode of procedure is the following:—A nobleman's family is committed to the care of a physician, who receives annually about 50*l.* for his attendance upon the superior members; while the servants, and the whole of the domestic department, are committed to a practitioner of less eminence, who is styled *house doctor*, and receives about a fourth of this sum. It is incumbent upon them both to be at the daily call of the persons committed to their care, and, as the fee is the same for attendance under all circumstances, they are generally well employed in their respective vocations. In cases of severe illness, (if any of the nobles are in such predicament,) the opinion of those whose constant attendance should warrant the conclusion that they are conversant with the constitutions of their patients is little attended to. Consultation is crowded upon consultation, and the family physician is merely a spectator in the scene. To these consultations, however, he will not much object, for the responsibility will be thereby divided. The consulting physician is paid at the rate of a guinea* or upwards per visit, by those at least who are in affluent circumstances. But there is all possible gradations as regards the scale of remuneration; generally speaking, however, the revenues of the first physicians in Russia are much lower than those of the same class in England.

Of all classes of the profession, accoucheurs are the most liberally remunerated, and they enjoy an advantage over all their colleagues, viz. that, by a species of habit, almost equal to law, they receive their fee when the patient leaves her chamber. Fifty pounds is the usual remuneration with the higher classes for an attendance which is in most cases nominal; for the accoucheur is in an adjoining room, and is never required to attend except in cases of danger or difficulty. All the attendance is intrusted to midwives, when none is in reality required. This fee also admits of great fluctuation, but an accoucheur of standing and reputation will not receive less than 10*l.* under most circumstances.

Where midwives are employed, it is usual for them to receive from 2*l.* to 5*l.* for their attendance, even from the mercantile classes, and much more from the nobility. The system adopted is frequently unfair to the regular medical attendant. He is often requested to be within call, but, unless there is danger, and the midwife demands his assistance, he receives no remuneration for the loss of his time or sleep. If he be called to the bedside, then he is entitled to the full fee; but this places him in the situation of being responsible for all the bad cases, and deprives him of the counterbalancing advantages of all the ordinary ones. Among the middle classes of society, these attendances are stipulated for in the annual salary, which seldom exceeds 14*l.* sterling.

As regards surgical operations, I have no information which I can venture to give as authentic upon this subject. All those of a minor order are not paid for separately from the annual stipend. I am inclined to believe that such operations as lithotomy, amputation, &c., when successfully performed, would be requited with a liberal hand by the higher classes.

The pay of medical men in government employ, whether civil or military, is upon too low a scale to ensure to the individuals even the necessaries of life. In the hospital establishments, the head physician receives only Ro. 1500, or about 65*l.* sterling per annum, with lodgings upon the premises, and the usual perquisites of fuel and candles. Such remuneration can ill requite the time which he is obliged to devote to

* Twenty-five roubles.

his duties; and were it not that, in general, a hospital physician has private practice, is attached to the court, and probably is a professor in the university, such pay would not entitle him to any respect or consideration. The visiting physicians, who act under his directions, have not the advantages which he enjoys of gratuitous lodgings, nor of the other perquisites; and they receive Ro. 800, or about half the sum of the superior. The service which they perform for this salary is a great demand upon their time.

The hospital mates (Felshers) receive nothing worthy of note; they are lodged and boarded in the hospitals.

With respect to the manner in which professional services in the universities are remunerated, some new regulations have just been issued. The salaries of all the professors are derived entirely from the crown; the students contributing nothing to the maintenance of the teachers. Their education is wholly at the expense of government. The following list of salaries, for the three principal universities, appears officially in the journals, dated 24th August, 1835.

| | St. Petersburg. | | Moscow. | | Harkoff. |
|---|---|---|---|---|---|
| Ordinary professor, | Ro. 5,500 | — | Ro. 5,500 | — | Ro. 4,500 |
| Extraordinary ditto, | 3,800 | — | 3,900 | — | 3,400 |
| Assistant ditto, . | 2,800 | — | 2,800 | — | 2,300 |
| Reader, . . . . | | | 1,800 | — | 1,600 |
| Demonstrator, . . . | | | 2,500 | — | 2,000 |
| Assistant, . . . . | | | 1,200 | — | 1,000 |

The professors enjoy the privilege of importing, free of custom-house dues, all foreign pamphlets and journals which are published abroad upon those subjects which they teach in the universities, and all such books are not under the control of the censorship.

There has been but little variation in the payment of medical men employed in government service since the year 1723, when the salary of those attached to the court was as follows:

| | | | |
|---|---|---|---|
| The Archiater, or Præses,* . | Ro. 3,000 silver | = | Ro. 12,000 |
| One body physician, . . | 1,200 | = | 4,800 |
| Two ditto, . . . | 800 | = | 3,200 |
| Surgeon, body, . . . | 600 | = | 2,400 |
| Professor, Anatomy, . . | 800 | = | 3,200 |
| Natural history, . . | 500 | = | 2,000 |
| Court physician, . . | 700 | = | 2,800 |
| Town physician, . . . | 400 | = | 1,600 |
| surgeon, . . | 150 | = | 600 |

It is difficult to ascertain precisely what is the value of situations about court in the present day. There are many perquisites attached to such situations, and their value can never be ascertained. The physicians in immediate attendance upon the imperial family are supposed to receive from ten to twelve thousand roubles per annum, (500*l.*)

The pay of the medical military staff is the same with that of the hospital civil staff and varies, according to rank, from 600 to 1,500 roubles per annum. This pay is quadrupled in active service, when out of the country.

There is a distinct class of court physicians. They perform active services alternately, and are obliged to remain the whole twenty-four hours in the palace. Each of these is called upon to serve about every fifth day. They receive 1,200 rubles (50 guineas) per annum as salary. They have no other duty than that which accident and emergency procure them, and are upon the spot to attend until other physicians arrive. The physician attached to the imperial family always resides in the palace which they occupy.

* President of the Academy of Medicine, and head of the faculty.

### III. MEDICAL SCHOOLS IN RUSSIA.

There are three principal universities in Russia proper, those of Moscow, Harkoff, and St. Petersburg; and each of these universities has a large school of medicine. Those of Moscow and St. Petersburg are the most considerable, but there is still a good school at Harkoff, situated in the southern part of the empire, and destined for the convenience of the natives of the conquered provinces on the Asiatic borders. Each university, which has a faculty of medicine, has also an institute of medicine of the same kind: the students who attend the latter are educated at the expense of government, and are subsequently obliged to serve six years, at least, in the civil or military service. The number of these students is fixed at one hundred for Moscow, and forty for Harkoff. This is, in reality, a kind of medical conscription.

On the present occasion I shall confine my observations to the chief medical school of the empire, that of St. Petersburg.

Although the first rudiments of a medical school in St. Petersburg are to be found in the establishment of the naval hospital, by Peter the Great, in the year 1715, yet the merit of organizing a medical faculty is due to Catherine the Second. In 1764, the Empress founded a medical college, which, with but few alterations from its first institution, is still recognized in the *Medico-Chirurgical Academy of St. Petersburg.* To the jurisdiction of this institution were subjected then, as at present, all the medical institutions, and all medical practitioners, with the exception of court physicians, in the empire. It was originally under the superintendence of a president, eight councillors, and a director general; but, in the year 1788, it was put under the control of a president, four physicians, and a secretary for the foreign department. These individuals appertained to the sixth class of nobility. There was also a head surgeon, of the seventh class, an operating surgeon, a surgeon's assistant, and a superintendent of the seventh class. The Academy formed a board of examination and control. All medical practitioners were subjected to the former, in order to obtain a licence to practise, and no other degree, either from Russian or foreign universities, exempted them from this ordeal. The Academy had the power of conferring medical rank, of fixing the rates of medical remuneration, and of assigning to the medical men employed by government their different posts throughout the empire. It exercised the privileges of conferring rewards and punishments, and of regulating the lazarettes and medical schools. It conferred the degree of doctor in medicine; thus assuming or usurping the privilege of an university. In the year 1789, a Russian Pharmacopœia was published by the Academy, which was at that period a supreme medical court.

Such was the institution as it was originally founded by Catherine; and the late *ukass,* published in 1835, regarding the same Academy, will prove that but little difference exists in its organization in the present day.

"The Medico-Chirurgical Academy of St. Petersburg (says this document,) enjoys the same privileges as the universities of the empire, and confers degrees in medicine, surgery, and in the veterinary art. It elects corresponding members for the diffusion of knowledge in the distant parts of the empire. It has its own censor, who inspects all manuscripts and publications of the members, and authorizes the translations of foreign medical works into the Russian language."

The Academy, as now constituted, consists of—

1. The president;
2. Two ordinary and two extraordinary professors;
3. Several assistants, viz. demonstrators, operators, manipulators, &c.
4. A general inspector;
5. A councillor, who presides over the supreme court of the Academy, with his secretaries.

The meetings of the Academy are styled *conferences,* and are attended by the medical professors only. In case an ordinary professor is prevented from attending, an extraordinary professor is allowed to act as a substitute. No official business can be transacted without the actual attendance of half the members. In cases of disagreement among the professors upon subjects of importance, the case is usually referred to the decision of the minister of the interior. The classes are regulated by the conference (Senatus Academicus), and the programmes of the different courses are determined by the respective professors. No one can be appointed professor who has not previously obtained the degree of doctor in medicine and surgery, unless some individual of great talent, and who has conferred some signal benefit on the faculty, be, from such circumstances, exempted from the necessity of these qualifications.

Lectures are delivered by the professors in the theatres of the Academy on the following subjects:

1. Natural philosophy and physics.
2. Natural history; comprising zoology, botany, mineralogy.
3. Chemistry, general and pharmaceutic.
4. Anatomy; combining a general view of comparative anatomy and experimental physiology.
5. Physiology.
6. General pathology.
7. Practical and theoretical pharmacy.
8. Materia medica, toxicology, and art of prescribing.
9. General therapeutics.
10. Surgery; including diseases and operations on the eye.
11. Midwifery; including diseases of women and children.
12. Medical jurisprudence.
13. Clinical medicine, surgery, and midwifery.
14. Literature; comprising the history of medicine, criticisms on the ancients, examinations in the Latin language, and dissertations on German and Latin classics.
15. General view of veterinary surgery, and minute description of epizootic diseases.

The lectures are delivered in the Russian and Latin languages, but the clinical lectures must be delivered in the Latin tongue. The lectures are said to be given daily, and are of two hours' duration; but, subtracting all the church holidays and other fête days, not more than three lectures a week can be considered as the average.

With all the advantages which the Academy offers to the medical student, (and, theoretically speaking, it must be allowed to be complete in all its parts,) it is singular how few Russian subjects arrive at the higher honours of their profession; almost all the professors and practitioners of eminence being graduates of foreign universities.

The following is a list of the present professors of the Academy, with the respective subjects taught by them.

| | |
|---|---|
| Professor Nechaeff, . | Mathematics, Physics, Chemistry. |
| Gorianinoff, . | Zoology, Mineralogy, Botany. |
| Bouialsky, . | Physiological and Pathological Anatomy. |
| Vellanski, . | Physiology, General Pathology. |
| Nelubin, . | Pharmacy. |
| Spasky, . | General Therapeutics, Pharmacology, Art of Prescribing. |
| Ilatiusky, . | Special Therapeutics. |
| Savinka, . | General and Special Surgery. |
| Seidlitz, . | Clinical Medicine. |
| Saloman, . | Diseases of the Eye. |
| Hotoffsky, . | Midwifery, Diseases of Women and Children. |
| Gromoff, . | Medical Jurisprudence, Hygiène. |
| Pelekin, . | Medical Literature. |

*Assistant Professors*, eight in number, repeat the lectures of the ordinary professors above mentioned, and are the demonstrators of practical botany, chemistry, anatomy, pharmacy, surgery, &c.

*Adjunct Professors.*

| | |
|---|---|
| Professor Sagoisky, . | Institutes of Medicine. |
| Schipusiusky, | Diseases of the Skin. |
| Yaktisky, . | Application of Bandages, &c. |

*Veterinary Professors.*

| | | |
|---|---|---|
| Professor Lukin, | . | Epizootic Diseases, and general Study of Veterinary Surgery |
| Sevetodoff, | | Comparative Anatomy and Physiology. |
| Prosoroff, | | Examination of Domestic Cattle. |

Upon the whole, it may be safely averred that the Russian medical student has every facility afforded him for prosecuting his studies to advantage, and for making himself master of his profession, as far as this can be accomplished by academical education.

The lectures commence on the 1st of September, and last till the 1st of July: hence the course is of ten months' duration. The examinations occupy the two months of *vacation*.

Five years' attendance on the lectures above specified entitle the student to examination for the degree of doctor in medicine. Four years' study are required from the veterinary student; three years from the apothecary.

There are three kinds of examinations continually in operation at the Imperial Academy:

1. The general public examinations of the students who have completed their studies. These take place during the two months' vacation.
2. Private examinations of individuals who apply for higher degrees, or of those who have been referred to their studies after the public examinations.
3. Examinations of foreigners, who apply for the licence of the Academy to practise any or every branch of medicine. Examination of graduates who aspire to higher honours.

Each professor is obliged to examine the candidate in that branch of medicine which he himself teaches; and of the capability of the student he makes report to the Senatus Academicus. All the professors present may question the candidate upon any subject, whether belonging to their own class or not. It is necessary that two professors and a secretary be present at each examination.

Russians are examined in the Russian and Latin languages. Foreigners have the choice of Latin, French, or English.

A preparatory classical education is demanded previously to the admission of students to the medical classes.

The medical majority is twenty-four years of age; and the degree of M.D. is not conferred before this period.

Beside these examinations for specific purposes, as degrees, &c., there is a general examination of all the students; an examination of probation, to ascertain the progress which they may have made in their studies. These are the examinations which take place after the termination of the lectures. It is from the result of this experiment that the subsequent division of the students into *classes of merit* takes place.

The report of the examining professors is laid before the Senatus Academicus, and the report is generally respected by the Conference, and serves for the arrangement of the four subsequent degrees; but, superior to this testimony of medical industry and capability of the student, as furnished by the professors, is the consideration which the Conference gives to the *moral conduct* of individuals, in preference to their acquirements in learning.

There are four degrees of comparison in the St. Petersburg Academy. The first is a degree less than the positive,—*goodish*, *good*, *better*, *best* Those who have passed their examinations with great credit are rewarded by medals, and by certificates of merit, signed by the Senatus Academicus.

Those who come under the first denomination are referred to their studies for another year, unless they are satisfied with the title which the fourth class affords them; for, as regards the class, this order must be inversed: the *goodish* is the last in rank, but first in position. Under this denomination are included those who have been prevented by illness from attending to their studies.

Those who are ranked in the third class, *good*, must remain a year longer in that class, to entitle them for promotion to the second class, *better*. These students are, however, generally satisfied with their honours, and for the most part are presented to the minister of the interior for service in government establishments.

Students guilty of improprieties of conduct are punished at the discretion of the president of the Academy; although a report must be made to the minister of the interior.

Government students—viz. such as are fed, clothed, and educated at government expense,—are compelled to serve gratuitously in different establishments for a certain number of years, regulated by the length of time they have been at the charge of government. Three years' service is demanded for one, five for two, and six for three years' gratuitous education at the expense of government.

The examinations of candidates for degrees is partly oral and partly in writing.

Those classed in the first and second order of students, as regards merit, are promoted to the rank of doctors of the first and second class, if they have obtained their degree with credit. Those of the third class in merit may, by remaining a year longer in the Academy, be promoted to the second class of doctors, after passing their examinations. If they do not aspire to this honour, they may take a degree in the third class of doctors, but are not doctors of medicine, unless, by giving proofs of superior talents in the exercise of their profession, they become entitled to apply for this honour.

Doctors passing an examination entitling them to the first rank receive medals and honorary diplomas, granted by the Senatus Academicus.

The Senatus Academicus selects yearly from the students who have passed their examination, one or two individuals, who are appointed to serve as supernumeraries in some hospital for the space of a year. At the end of this probation, they are supplied with funds to enable them to travel abroad, and visit foreign universities, for the completion of their education, and for the purpose of improving their own institutions. These *travelling fellows* are obliged, upon their return home, to serve for the space of three years in some large hospital, and are then eligible to be elected professors in the Academy.

Students who have not made such progress in their studies as may allow them to take a degree in any of the classes, are distributed among the hospitals, bearing the title of *candidates of medicine*, for the space of one or more years. During this period they must attend lectures, and act as dressers and assistants to senior officers. Upon receiving certificates of good conduct, and having given sufficient proofs of advancement in their studies, they may subsequently be made doctors of the third class; or, if willing to submit to another course of examination, may be promoted to the second class.

Foreigners, who have studied in other universities, may claim the same privileges as native students, by submitting to different tests and examinations, and may be ranked in the first, second, or third class, as they prove themselves worthy of the respective honours. No honours, degrees, or licences from foreign universities exempt strangers from this course of examination. Some few, however, have occasionally been exempted, through imperial favour. Foreigners, whose sole object consists in possessing a licence to practise, generally content themselves with the lowest degree, unless they look forward to professorships or hospital appointments.

As regards the army or navy, there is no particular plan of education; the students receive all the same education.

Upon an average, sixty students graduate annually in the St. Petersburg Academy. Few of these take the degree of doctor in medicine and surgery.

There is a class of practitioners who have received permission to practise, by particular favour, without previous examination; and, as they do not come under any of the academical titles, they receive that of *Practicant.*

I subjoin the examination of an English candidate, which may serve as a fair sample of the nature of the examinations in general. [See *Appendix*.]

Two medical periodicals are published under the authority of the Academy,—the Military Journal, which appears at irregular intervals, and the Weekly Gazette of Health. Both of these publications are in the Russian language. The profits arising from the sale of the military journal are devoted to medical charities, and all medical men in government service are obliged to subscribe to it.

Although the medical school of Moscow is but a branch of the St. Petersburg Academy, yet the university of Moscow has its medical faculty; a privilege which the university of St. Petersburg does not enjoy. The establishment of a medical faculty *per se,* independently of the university, was an innovation of the president, Sir James Wylie.

Besides the establishment for lectures, the St. Petersburg Academy possesses the following collections and institutions:—

1. An extensive Library. 2. A Cabinet of Natural History. 3. A Chemical Laboratory. 4. A Cabinet of Minerals, (very rich.) 5. An Herbarium. 6. A Zoological Cabinet. 7. A Cabinet of Anatomy. 8. A Cabinet of Pathology. 9. A large collection of Models in wax, and of Surgical Instruments, &c. 10. A Dispensary, and Pharmaceutic Laboratory. 11. Clinical Wards for Diseases of the Eye. 12. An Hospital for the Inmates of the Academy. 13. A Veterinary Hospital.

Foreign journals, from all parts of Europe, are received by the secretary, who makes such extracts from them as he deems necessary, and these, translated into Russian, are inserted in the two periodical journals before mentioned.

### IV. HOSPITALS IN RUSSIA.

Although few countries can boast of finer institutions for the sick and infirm than Russia in the present day, it has nevertheless been the work of nearly two centuries to bring them to their actual state of perfection. Commenced, as we have seen, by a private individual, whose example was soon followed by government, they only began to have a character of importance under Peter the Great. But it is with these as with all public institutions,—it is the slow and improving hand of time alone which can rectify what is amiss, and consolidate what is good: hence, as the method of treating diseases has varied with the acquirement of knowledge, so has the hospital patient reaped the fruits of such advantages as have been offered by the progress of medical improvements.

As regards the external appearance of the hospitals in Moscow and St. Petersburg, they are splendid in the extreme, resembling more the palaces of princes than the abodes of the sick. But it is of their discipline we have to speak, and many of the regulations of these institutions are worthy of imitation by similar establishments in other parts of Europe. One great advantage which they possess over charitable institutions of a similar kind in England, is the *daily* admission of patients; the vacant beds being immediately occupied by the most urgent cases.

All applicants are not admitted indiscriminately into the General Hospitals; for there are others devoted to the reception of particular diseases, as eruptive fevers, venereal diseases, &c.; and persons labouring under diseases which are considered incurable are not treated in the hospitals, but are admitted into almshouses.

The time of admission is from nine to twelve o'clock, when the patients must present themselves in the receiving room. Here they are inspected by the house-physician, who selects such cases as appear to him the most interesting. This selection, however, is not final, for it must be approved of by the superintendent. When the choice is confirmed, the patient is immediately conducted into a bath-room, where he is well washed; and all his clothes are taken from him, and are deposited in a wardrobe, to be restored to him when he quits the hospital. He is immediately supplied with other garments,—viz. a linen shirt, a pair of drawers, stockings, a nightcap, a pocket handkerchief, a pair of slippers, and a loose morning gown of woollen or linen, according to the season. Thus attired, he is conducted to his bed, and is soon visited by the physician of the division in which he is placed. His bedstead is of iron, furnished with two mattresses, a pair of linen sheets, and a blanket. He quits his loose gown when he gets into bed; and this is rolled up like a soldier's cloak, and placed at the foot of the bedstead upon a little stool. By the side of his bed is a small table with a drawer, which contains his spoon, his knife, his salt, &c.

Over the patient's head is suspended a sheet of paper, with the name, the age, the owner of the individual, (if a slave,) the day of admission, the duration and name of the disease. The sheet is divided longitudinally into four divisions. The first column shows the *decursus morbi,* under which head the physician states daily, in Latin, the progress of the disease, the different changes which have occurred, and the effects of remedies. The second column contains the prescriptions of the medicines which have been administered internally. The third shows the external applications which have been employed. The fourth prescribes the diet. This paper remains continually at the bed-head, but its contents are copied into a book, which must be countersigned by the superintendent before the apothecary is allowed to make up the prescriptions.

According to the urgency of the symptoms, the patient is visited once or twice daily; and, as a resident medical man is always within call, so he is never without help in need.

The linen is changed frequently; it being left to the judgment of the physician to have it changed as often as he deems it necessary. The food is of the best quality, and the quantity is regulated by the medical officer.

The medicine is administered by the *felshers,* whose duty it is to give it at the times prescribed, and to *oblige* the patients to swallow it; not leaving it to their option to accept or reject it.

When desirous to leave the hospital, the patient is furnished with an order authorizing his dismissal, either because he no longer requires medical assistance, or because he finds the confinement irksome. He can leave at all times, upon applying for his release; but, if he quit before his cure be effected, or contrary to the advice of the medical officer, he is not again received.

As regards the internal arrangement of the hospitals, there is no cause for complaint. The wards are spacious and lofty, the beds not too much crowded together, and cleanliness is carried to a point almost deserving of ridicule. The different offices attached to the establishment,—as the bakehouse, kitchen, laundry, pharmacy,—are all perfect in their kind; and, as they are liable to be visited by the authorities at all hours, so there is a moral necessity for their good administration. The baths are very superior, and of various kinds. There are baths for mere ablution, vapour baths, and simple and medicated baths of all descriptions, always in readiness.

The number of medical officers attached to each hospital will naturally vary with the number of patients therein contained; but each hospital has its superintendent physician, whose duty it is to approve of the choice made of the patients before they are admitted; to sign the order for the medicines delivered out of the Pharmacy, and to perform various duties regarding the internal police of the establishment.

There are always from two to three visiting physicians, who regularly visit the patients once, and often twice a day; and there is a physician always residing on the premises, ready for any emergency. Each medical officer has his attendant felsher, who accompanies him in his rounds, and receives his orders for the day. The men are attended by nurses of their own sex, and the women by theirs. They are divided into night and day watchers, and there are others appointed to watch the watchmen. There is also a matron.

The wards are fumigated daily by burning vinegar upon hot bricks, upon which are sprinkled some aromatic herbs. Water-closets, a luxury unknown to the nobility twenty years ago, are now furnished to every hospital.

The most decided defect in all these institutions is a thorough disregard to ventilation; for fumigation is not ventilation, nor is the disguising a disagreeable smell by the overpowering influence of a more supportable scent, a compensation for the want of fresh air. But the habits and customs of a people, influenced or formed perhaps, as they have been, by the rigour of their climate, must be taken into consideration; and we cannot expect to find in a hospital what exists in no other habitation. The Russians, of all people in the world, most dread the effects of cold air admitted into their houses. They brave the external frost and snow with impunity; but, when once the Russian takes off his furs, and enters his house, he trembles at the idea of an open window. He can only live in an atmosphere of a certain temperature, and must be guarded from the influence of every breath of air. He consults his thermometer, and, unless he finds it from 15° to 17° of Reaumur, (65° to 70° Fahr.,) he feels chilled and uncomfortable. "Heat breaks no bones" is a Russian proverb, quoted as an apology for a suffocating atmosphere. The thorough airing of a room by throwing up the sash, and the smell of fresh morning air, so essential to the comforts of an Englishman, have nothing to recommend them to the inhabitants of the North. If this, then, be the case in private houses, can it be supposed that a different system should be adopted in public hospitals? Such an innovation would bring them more into disrepute with the patients than the certainty of being anatomized after death. It would alarm a physician of the Edinburgh Infirmary, who is accustomed to find the windows of his fever-wards open in the month of January, to see his patients stewed in a ward heated to 16° Reaumur. I have conversed upon this subject with some of my countrymen, and they inform me that they have often tried the experiment of admitting fresh air into their patients' rooms; but in vain. It is useless to strive against deep-rooted prejudices in matters which constitute the chief comfort of a people. It is so in health; it is more so in disease: heat they will have, if it is to be procured. In spite of their obstinacy, I have every reason to believe that it is very injurious to them, and that their convalescence is often protracted from this cause alone. I am convinced that this system is productive of a variety of ailments and uncomfortable sensations in those who call themselves in health. Headaches, lethargy, hemorrhoidal affections, are the lot of the most healthy. In a frame still more debilitated by severer diseases, I am certain that the effects of heat are most pernicious. In private practice, I have often found the most beneficial results when I have been able to overcome prejudices, and admit fresh air into the sick room, or remove the patient into a more spacious and better ventilated apartment. It is not often that I have succeeded in my attempts; and nothing is more irksome to a medical man than the feeling that the small means in his power are not turned to the best account, and that, whilst he finds no obstacle to the administration of medicines which are of the most doubtful efficacy, he is often prevented from using such means as are unequivocally advantageous. In cases of scarlatina, a disease so prevalent and so destructive in St. Petersburg, I have had to cope with furs, blankets, warming pans, and 18° of Reaumur; nor have I been able to succeed in my attempts to allow the introduction of a little fresh air, or to administer to the sick a little cooling beverage.

With these and some other trifling exceptions, there are no hospitals in Europe better conducted than those of Russia. Food, clothing, comfortable lodging, humane treatment, and the best medical advice, are at the disposal of the sick. I have myself sent many poor patients to the hospitals; many have gone reluctantly, but never has it occurred to my knowledge that the individual sent has returned with the same prejudices which almost prevented his going into the institution.

There are four kinds of diet prescribed for the sick in the Russian hospitals by the attending physicians. The following is the dietary of the Galitzin hospital, the richest of its kind in Moscow.

The *full diet* is soup containing barley or groats; a pound of beef, (twelve ounces avoirdupois;) half pound of roast meat; two pounds of brown bread, or a pound and a half of white bread. The *ordinary diet* consists of the above, with the exception of the roast meat. The *low diet* consists of a basin of chicken or veal broth, with a portion of fowl, and a pound of white bread. The *extraordinary diet* consists of half a fowl, and a pound of white bread. The *drink* is composed of *kvas* and barley-water. Wine is prescribed, if necessary.

The diet of the great town hospital is thus regulated:

*Full diet.* Cabbage soup, one pound of beef, with boiled buckwheat, a pound and a half of bread. *Moderate diet.* Soup with groats, a pound and one-eighth of bread. *Low diet.* Veal or chicken broth, a pound of white bread. To this is added, as may be judged proper, the half low diet, consisting of weak broth, fish soup, groats with milk, jelly, &c. According to circumstances, also, fowl, milk, apples, prunes, salted cucumbers, beer, and wine are administered to the sick.

The fasts prescribed by the Russian church are observed by the hospitals, unless under particular circumstances, when it is at the discretion of the medical attendant to forbid the observance. The fast diet consists of a pound and a half of mashed potatoes, turnips, or carrots, half a pound of fish twice a week, half a pound of groats, three-quarters of a pound of bread, for dinner. For supper, one pound of mashed potatoes, three-quarters of a pound of bread, a pint of kvas,* or beer.

### HOSPITALS IN MOSCOW.

How much the utility of hospitals has been appreciated in Russia, is evident from the extent to which they have multiplied. Moscow, which two centuries ago barely reckoned a single institution of this kind, has now to boast of seventy charitable establishments for the relief of the sick poor. Some of them are of minor importance, but some are of gigantic magnitude. They are supported by government, by voluntary contribution, and by private endowment. The following table contains a view of the seven largest hospitals of this city, in the year 1834. It may be well to premise, that the population of Moscow, in 1833, was 333,260.

| Names of Hospitals. | Number of Beds. | Number of Patients. | | Cures. | Deaths. |
|---|---|---|---|---|---|
| | | Males. | Females. | | |
| Poor's Hospital ......... | 244 | 1,582 | 1,549 | 2,488 | 441 |
| Paul's ...................... | 176 | 1,485 | 403 | 1,528 | 199 |
| Galitzin .................... | 145 | 513 | 353 | 736 | 130 |
| Sheremetief......... ..... | 68 | 468 | 425 | 714 | 116 |
| Catherine.................. | 220 | 1,179 | 604 | 1,327 | 242 |
| Town........................ | 450 | 2,203 | 881 | 2,430 | 503 |
| Military.................... | ...... | 12,256 | 963 | 11,542 | 1,204 |

An idea may be formed of the expense and general establishment of these hospitals from that of the Galitzin, which is as follows:

Medical establishment:—One superintendent; one physician in chief; one assistant physician; eight physicians in ordinary; one midwife; twenty-four felshers, or dressers; one apothecary; one assistant apothecary; one chaplain; ninety male and twelve female nurses.

The annual cost of this hospital is estimated at 180,000 rubles, or 8000*l.* sterling.

*Hospital for Diseases of the Eye.* This establishment, being supported by voluntary contribution alone, is not upon a very large scale. It contains, however, fifty beds, and has an operating theatre and a consultation room. It is computed that about five hundred patients are admitted annually into the wards, and that from fifteen to sixteen thousand are treated as out-patients. There are, upon an average, three hundred operations performed annually upon the eye; and of these, from fifty to sixty are for cataract.

*Orthopœdic Institution.* This is a private speculation, conducted by Dr. Mandileny. It possesses the advantage of a gymnastic school, which in fine weather is held in a large garden, and in winter in a large hall, heated to a proper temperature. This school is frequented by young persons, for the sake of exercise, as well as by such patients as are supposed likely to benefit from the different kinds of exercises. The bath department is very complete, and is found very serviceable in the treatment of many distortions.

*Hospital for the Insane.* This was instituted in 1791, but it is not of very considerable extent. The building consists of two stories; each is divided, through its whole length, by a spacious hall, which serves as a dinner-room and a promenade in inclement weather. The bed-rooms, or rather the cells of the patients, are situated on each side. The women are lodged below, the men above.

Upon the whole, this is one of the worst organized establishments in Moscow, and is much inferior to that in St. Petersburg. The space is not sufficient to allow a proper separation of the patients. It is in reality a *hospital*, providing for physical rather than moral ailments, and should be considered as a section of an establishment rather than a complete institution.

* The drink of the common people. It is made with rye-flower and rye-malt, fermented with yeast.

The food is not so liberally distributed as in other hospitals. The full diet consists of two pounds of bread, two-thirds of a pound of meat, with boiled buckwheat or barley. The drink is kvas. The clothing is good and appropriate. There are, upon an average, 150 patients in the hospital.

*The Foundling Hospital.* It is not within the scope of these observations to enter into the disputes regarding the utility or impropriety of foundling hospitals; but, in presenting a sketch of the hospitals in general, as they are at present conducted in Russia, it is necessary to include these in the list. Moscow boasts of the finest institution of this kind in Europe. It is not used as a receptacle for illegitimate children only, but is open to the poorer classes who are not able to maintain their offspring. In this respect it differs from many institutions in Europe, and is designated the Imperial House of Education.

It was founded by the Empress Catherine in 1762, and was designated House of Education, from its being open not merely to the children found in the box at the gate, but because mothers, unable from poverty to maintain their children, are allowed to enter to nurse their own offspring in the institution, and are allowed a nurse's salary during the time they remain there. This circumstance was probably unknown to the philanthropist Allen, who, when this noble edifice was pointed out to him by the late Emperor, instead of expressing admiration, recommended its immediate demolition.

The external appearance of the building is magnificent. It is situated upon the banks of the Moskwa, and is approached at one time by a long avenue of trees, and a large garden is attached to it. The whole establishment is upon a scale of great magnificence; the wards are lofty and spacious, and all the offices are commodious in the extreme. Neatness and cleanliness prevail in every department. The hospital contains three thousand inmates of all ages. Every foundling has its little cradle placed by its nurse's bedside. The mother has not only comforts but luxuries afforded her, and consequently few are the beds which are allowed to remain long vacant. Women of the very refuse of the people, half starved and in rags, find food and raiment, and pecuniary remuneration, for the bare trouble of solicitation. The building is divided into a variety of wards, to suit the different ages of the children, and the system of education which is adopted at different periods. The ground-floor is occupied by the offices and the reception-room for the infants. There is a chapel for baptism, which ceremony is performed as soon as they are admitted. They are received at all hours, and none are refused except the children *des officiers supérieurs.* The children of private soldiers are admitted.

Seven thousand children are received annually, upon the average, in this institution. A ticket is attached to the child's neck upon admission, and a duplicate is given to the person who leaves the infant, so that it may be reclaimed afterwards by the parents, if they request it.

According to the different talents which these children manifest, they are divided into different classes for education. Some are taught the arts, sciences, and living languages, and are subsequently sent to the universities, where they take degrees in medicine, or become tutors and teachers. The females are educated for governesses, and serve as such in families who reside in the interior of the empire; but a governess educated in the Foundling Hospital is not allowed to reside in St. Petersburg or Moscow in that capacity. The children who do not aspire by their talents to such high situations, are taught trades, and are apprenticed to tailors and shoemakers, milliners and mantua-makers. Such as are incapable of any application are maintained in a separate establishment, at the expense of the hospital.

This charity also extends its bounties to those who apply for assistance to nurse their children at home, in preference to placing them in the hospital. Many of the children are sent into the country after having remained a certain time in the hospital, and are brought up by peasants, who, if they have no children of their own, are allowed to adopt them. At the age of eighteen, the boys are transported to the villages which belong to the crown, and are employed as labourers. A colony of these children has been established in the government of Smolensko. When the education of such as have been brought up in the institution is completed, a passport is given them as free subjects, and they can never become serfs under any pretext.

Two infirmaries are attached to the institution; one containing 128 beds for the children, and one of twenty beds for the attendants when ill. There is also a hospital of forty-four beds, established for what a witty author has styled the *second chances.* Women are allowed to find succour here without divulging their names: no questions are asked. Midwifery is taught females in this institution.

There is a country residence belonging to the establishment, where the children are sent in the summer, for the benefit of the air.

Although 7,000 children are said to be admitted annually, still the number of living foundlings, in Moscow and its environs, in 1832, amounted only to 21,287, out of all who had in a long series of years been received into the establishment.

Sixteen physicians are attached to the Foundling Hospital.

### HOSPITALS IN ST. PETERSBURG.

The following table, besides the names and extent of the principal hospitals of St. Petersburg, exhibits the number of patients admitted during the year 1835, and the general result of their diseases.

| Hospital. | Number of Beds. | Patients admitted. | | Died. | |
|---|---|---|---|---|---|
| | | Males. | Females. | Males. | Females. |
| 1. Aboukoff | 454 | 3,424 | 1,425 | 515 | 174 |
| 2. Mary Magdalene | 160 | 1,426 | 416 | 273 | 73 |
| 3. Marie { In-Patients | 330 | 2,537 | 1,125 | 742 | 297 |
| | | | | *cured* | *cured* |
| 3. Marie { Out-Patients | | 33,007 | 14,937 | 21,878 | 6,992 |
| 4. Imperial Guard Lazaret | | 8,498 | | 304 | |
| 5. Military Hospitals | | 24,554 | | 1,414 | |

*Hospitals in Odessa.*

| Military Hospital. | Remaining. | | Admitted. | | Cured. | | Died. | | Remaining. | |
|---|---|---|---|---|---|---|---|---|---|---|
| 1834 | 198 | | 1,590 | | 1,538 | | 86 | | 164 | |
| 1835 | 164 | | 935 | | 905 | | 57 | | 137 | |
| Civil Hospital. | Males. | Females | M. | F. | M. | F. | M. | F. | M. | F. |
| 1834 | 217 | 137 | 1,782 | 646 | 1,552 | 594 | 236 | 95 | 211 | 94 |
| 1835 | 211 | 94 | 1,693 | 478 | 1,483 | 413 | 205 | 67 | 206 | 92 |

*Archangel Hospital.*

| Beds, 480 | Remaining. | Admitted. | Cured. | Died. | Remaining. |
|---|---|---|---|---|---|
| 1833 | 208 | 2,812 | 2,810 | 108 | 102 |
| 1834 | 102 | | | | |

*Cronstadt Military and Naval Hospital.*

| Beds, ordinary No. 2000,* | Remaining. | Admitted. | Cured. | Died. | Remaining |
|---|---|---|---|---|---|
| 1834 | 1,715 | 24,834 | 23,712 | 1,334 | 1,503 |
| 1835 | 1,503 | | | | |

* By converting adjoining buildings into lazarets, the number of beds may be increased to 3,000, as occurs often in the spring.

### APPENDIX.

*Examination of a Medical Student, before the Imperial Medico-Chirurgical Academy of St. Petersburg.*

*Professor of Anatomy and Surgery.*

What is the structure of the heart?
stomach?
eye?

What are the different dislocations of the hip-joint? How are they distinguished, and what are the modes of reducing each kind?

What is the structure of the testicle? Describe the operation of extirpation of the testicle.

*Professor of the Practice of Physic.*

What are the symptoms and nature of epilepsy? What are the different supposed causes of its production? What are the most approved methods of treatment? What are the symptoms of delirium tremens?* What influence has intoxication upon the functions of the brain?

Describe the symptoms of ophthalmia, and relate the history of the Egyptian ophthalmia.

*Professor of Physiology.*

The student had to write a Latin essay on sleep and wakefulness, in the presence of the professor.

*Professor of Chemistry and Pharmacy.*

What is the composition of the atmosphere? Give some practical illustration of the expansion of air by caloric. What is the composition of the blue pill?

*Professor of Physics.*

Describe the principles upon which the barometer and thermometer are constructed.

*Professor of Botany.*

What is the class and order of the digitalis? What are its medical properties?

*Professor of Natural History.*

From whence is castor derived? Describe the animal. What is rock salt?

*Professor of Midwifery.*

Describe the membranes of the fœtus in utero, and give Dr. Hunter's account of the same.

These examinations occupied five successive sittings, each of about half an hour's duration.

One peculiarity exists in the examinations. It is customary to question a student upon some celebrated physician's opinions upon particular subjects. Thus, a professor demands what is Sir Astley Cooper's opinion regarding the union of bones fractured within the capsular ligament? Does the neck of the thigh-bone, when so fractured, ever unite? What was the late Dr. Frank's (of Vienna,) favorite remedy in such or such diseases? What does Hufeland employ as a favorite remedy for ———? These questions are frequently put, and puzzle foreign students: they are more applicable to students of their own universities, because the works of these men are put into their hands as class-books.

As the licence granted to those who have passed such an examination as the preceding allows them to practise generally, few comparatively take the degree of M.D.

Those who are examined previous to entering the military service are obliged to perform operations upon the dead subject in the theatre. An English student, who had passed all his previous examinations with eclat, was desired to amputate a leg: he commenced his incision without previously applying the tourniquet. He was remanded for six months, and passed afterwards.

G. L.

*St. Petersburg; December,* 1836.

* A most common disease in Russia.

